# Evolution of the eukaryotic ARP2/3 activators of the WASP family: WASP, WAVE, WASH, and WHAMM, and the proposed new family members WAWH and WAML

**DOI:** 10.1186/1756-0500-5-88

**Published:** 2012-02-08

**Authors:** Martin Kollmar, Dawid Lbik, Stefanie Enge

**Affiliations:** 1 Abteilung NMR basierte Strukturbiologie, Max-Planck-Institut für Biophysikalische Chemie, Am Fassberg 11, D-37077 Göttingen, Germany

**Keywords:** ARP2/3 activation, WASP family proteins, WASP, WAVE, WASH, WHAMM

## Abstract

**Background:**

WASP family proteins stimulate the actin-nucleating activity of the ARP2/3 complex. They include members of the well-known WASP and WAVE/Scar proteins, and the recently identified WASH and WHAMM proteins. WASP family proteins contain family specific N-terminal domains followed by proline-rich regions and C-terminal VCA domains that harbour the ARP2/3-activating regions.

**Results:**

To reveal the evolution of ARP2/3 activation by WASP family proteins we performed a "holistic" analysis by manually assembling and annotating all homologs in most of the eukaryotic genomes available. We have identified two new families: the WAML proteins (WASP and MIM like), which combine the membrane-deforming and actin bundling functions of the IMD domains with the ARP2/3-activating VCA regions, and the WAWH protein (WASP without WH1 domain) that have been identified in amoebae, Apusozoa, and the anole lizard. Surprisingly, with one exception we did not identify any alternative splice forms for WASP family proteins, which is in strong contrast to other actin-binding proteins like Ena/VASP, MIM, or NHS proteins that share domains with WASP proteins.

**Conclusions:**

Our analysis showed that the last common ancestor of the eukaryotes must have contained a homolog of WASP, WAVE, and WASH. Specific families have subsequently been lost in many taxa like the WASPs in plants, algae, Stramenopiles, and Euglenozoa, and the WASH proteins in fungi. The WHAMM proteins are metazoa specific and have most probably been invented by the Eumetazoa. The diversity of WASP family proteins has strongly been increased by many species- and taxon-specific gene duplications and multimerisations. All data is freely accessible via http://www.cymobase.org.

## Background

Eukaryotes mediate many cellular processes, including phagocytosis, intracellular trafficking, cell division, and cell locomotion, by remodelling of the actin cytoskeleton [1-4]. In many cases, signals from Rho family G-proteins activate proteins of the WASP family (WASP, Wiskott-Aldrich syndrome protein), which stimulate the actin filament branching activity of the ARP2/3 complex [5] resulting in specialised networks of actin filaments [6,7]. The ARP2/3 complex consists of two actin-related proteins, Arp2 and Arp3, and five additional subunits [5,8]. The ARP2/3 complex alone is very inefficient in nucleating actin filaments but becomes activated when binding to the sites of existing actin filaments. In addition, it needs to bind to so-called nucleation promoting factors (NPFs). NPFs of the WASP family belong to class I NPFs and have characteristic VCA domains at their C-termini consisting of one or more WH2 domains (V; WASP homology 2 domain, also known as verprolin homology domain), a central domain (C; also known as cofilin homology or connector domain), and an acidic (A) C-terminal domain. While the WH2 domains bind G-actin the CA domains bind to and activate the ARP2/3 complex.

The founding member of the WASP family was the WASP protein, which is deficient in Wiskott-Aldrich syndrome. The Wiskott-Aldrich syndrome is an X-linked human genetic disorder that leads to a variety of defects in the immune system, including thrombocytopenia, compromised immune function, and susceptibility to leukemias and lymphomas [9-11]. In a genetic screen for second-site suppressors of a mutation in one of the cAMP receptors, another protein that showed significant homology to WASP, called Scar, was identified in *Dictyostelium discoideum *[12]. At the same time the so-called WAVE-1 protein (WASP-family verprolin-homologous protein-1) was identified in a database search for proteins with sequence homology to the VCA region [13]. The name Scar has subsequently been used for the *Dictyostelium *and the plant homologs, while WAVE has been used for all metazoan family members.

WASP proteins are multi-domain proteins consisting of an N-terminal WH1 domain, a basic domain containing many lysine and arginine residues, a GTPase binding domain (GBD) consisting of a CRIB motif (for Cdc42/Rac interactive binding) followed by an autoinhibitory domain, a poly-proline region, and the VCA domain. The WASP protein by itself is inactive through intramolecular binding of the C domain to the autoinhibitory domain [14]. Binding of G-proteins to the GBD domain releasing the intramolecular link activates the WASP proteins. A comparison of proteins of the WASP and the WAVE family shows that WAVE shares many of the WASP domains, including the polyproline rich region and the VCA domain. Unlike WASP, WAVE family members do not contain a GBD domain and they have a novel protein region instead of the WH1 domain, termed WAVE homology domain (WHD). WASP forms a constitutive complex with WIP (WASP interacting protein), which binds to the WH1 domain and has been shown to stabilize WASP [15]. Three WIP homologs have been identified in the human genome [16], and all seem to be able to bind to the two WASP homologs. In contrast, WAVE proteins form a 400 kDa heteropentameric assembly called the WAVE regulatory complex that consists of WAVE, a protein from the CYFIP family (e.g. SRA1), NAP1, ABI, and HSPC300 [17,18]. The three mammalian WAVE homologs seem to participate in the same protein complex and thus differences between them are likely to be at the level of tissue distribution [17].

In the last years, several further proteins have been identified that also activate the ARP2/3 complex like WASH (Wiskott-Aldrich syndrome protein and Scar homolog) [19], WHAMM (WASP homolog associated with actin, membranes, and microtubules) [20], and JMY [21], which is a WHAMM homolog [22]. Still, there is only limited knowledge about their structure, sequence homology, and phylogenetic distribution. Early analyses have revealed that WHAMM might be restricted to vertebrates [20], while WASH seems to be more widely distributed [19,22]. Like WAVE, WASH functions in a heteropentameric complex containing Strumpelin, FAM21, SWIP, and CCDC53, which show some sequence homology to the corresponding proteins of the WAVE complex [23].

Very recently, the genomes of some representative eukaryotes have been analysed with respect to their WASP family protein content [22]. However, the analysis of motor proteins in sequenced insects [24] has shown that it is essential not only to analyse the genomes of representative members of the main eukaryotic taxa but to also analyse the genomes of very closely related species, because not even those might contain the same set of homologs. In addition, it is essential to search directly in the respective genome sequences and to manually reconstruct potential homologs because datasets of predicted proteins are far from being complete. In those automatically generated datasets many proteins are missing at all, and most of the predicted proteins contain mispredicted intronic sequence and miss exons thus hindering the identification of all homologs. Especially in the case of the WASP family proteins, that contain many very short domains and sequence motifs, mispredicted exons easily result in missing domains and thus misleading hypotheses about potential physiological functions. Here we performed a search for WASP family proteins in the available genome assemblies of more than 400 eukaryotes. All sequences were identified at the genomic DNA level and the genes reconstructed manually.

## Results

### Identification and assembly of WASP family proteins

The WASP family proteins contain many but very short sequence motifs and domains. These domains are not only characteristic for WASP proteins but are also part of proteins from other families like the WHD domain, that is shared by the Nance-Horan syndrome protein (NHS), the WH1 domain, that is shared by the Ena/VASP protein family, the WH2 domain, that is contained for example in Spire, Cobl, missing-in-metastasis (MIM), and WIP, and the CA domains, that are shared for example by certain coronins and class-1 myosins. Given such short sequence motifs a few missing residues could prevent their identification and lead to a wrong classification or even removal from the dataset. Vice versa, the identification of just one or two of the domains contained in one of the WASP family proteins and subsequent thorough analysis of the genomic region could lead to the identification of so far unrecognized or very divergent members of the WASP family. While this subfamily categorization is mainly based on domain organisation patterns, the classification of the duplicated WASP family genes into subgroups is based on the phylogenetic analysis of the corresponding protein sequences. Thus, it is of major importance to obtain the best sequence data possible and to create the most accurate sequence alignment. We had to manually assemble and annotate all WASP protein family sequences used in this study mainly for two reasons. A) Automatic gene predictions are error-prone and even those gene predictions are only available for a small subset of all sequenced eukaryotic genomes [25]. B) Because all WASP family genes encode long unstructured and low-complexity regions (for example the poly-proline regions), and the acidic C-termini (the A domain, also of low complexity) are often encoded by a single exon, the automatic gene predictions contain many mispredicted exons/introns and unrecognised N-terminal/C-terminal parts. Most likely due to the long low-complexity regions, only a few full-length mRNA/cDNA sequences for WASP family proteins are available. If partial cDNA/EST clones were found then those encoded in most cases the N-terminal WH1, WHD, WAHD, or WMD domains. The eukaryotic genomes were analysed in an iterative search process, meaning that those genomes, for which the WASP family proteins could not unambiguously assembled in the first instance, were reanalysed as soon as further data was added to the multiple sequence alignments. In this way the completeness of the search for WASP family proteins and the accuracy of the gene assembly and annotation have continuously been re-evaluated and improved. In addition to manually assembling all sequences, the multiple sequence alignments of the WASP sequences have been created and were maintained and improved manually (Additional file 1).

The WASP dataset contains 1021 sequences from 408 organisms (Table 1). 848 sequences are complete and an additional 58 sequences are partially complete. Sequences, for which a small part is missing (up to 5%), were termed "Partials" while sequences, for which a considerable part is missing, were termed "Fragments". This difference has been introduced because "Partials" are not expected to considerably influence the phylogenetic analysis. WASP family genes were termed pseudogenes if they contain more disablements like frame shifts and miss more conserved sequence regions than can be attributed to sequencing or assembly errors. The different WASP subfamilies are distinguished by their domain organisations outside the common proline-rich and C-terminal VCA domains.

**Table 1:** Data statistics

	WASP	WAVE	WASH	WHAMM	WAWH	WAML
**Sequence**						

Total	400	344	165	76	19	17

From WGS	375	332	148	68	17	17

Domains	7	3		2	5	2

Amino acids	207219	251639	78326	58689	14066	8614

Total pseudogenes	3	19	4			

Pseudogenes without sequence	1	2	1			

						

**Completeness**						

Complete	316	312	138	47	18	17

Partials	33	9	9	7		

Fragments	50	21	17	22	1	

						

**Species**						

Total	316	169	158	42	11	3

WGS-projects	275	155	139	34	9	3

EST-projects	87	69	59	17	4	1

WGS- and EST-projects	128	87	81	23	6	2

### Classification and analysis of the WASP protein family

**Figure 1:**
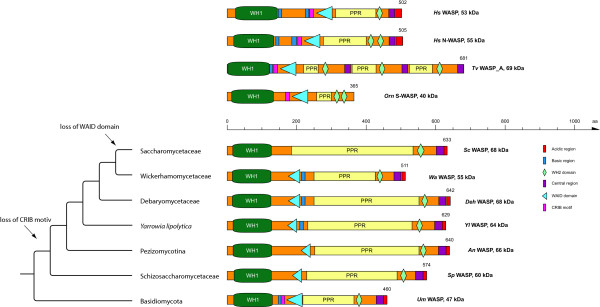
**Domain organisation of representative WASP proteins**. A colour key to the domain names and symbols is given on the right except for the WH1 domain and the poly-proline region (PPR) that are coloured in dark green and yellow, respectively. The abbreviations for the domains are: WH2, WASP homology 2 domain; WAID, WASP autoinhibitory domain. The phylogenetic tree on the left shows the schematic branching of some major taxa of the Dikarya fungi and, as single representative, *Yarrowia lipolytica*. For each branch, the domain organisation of one representative WASP homolog is shown on the right. The schematic tree has been created based on the most comprehensive phylogenetic analysis of yeast species [26]. Species abbreviations: *Hs *= *Homo sapiens*, *Tv *= *Trichomonas vaginalis*, *Orn *= *Oreochromis niloticus*, *Sc *= *Saccharomyces cerevisiae*, *Wa *= *Wickerhamomyces anomalus*, *Deh *= *Debaryomyces hansenii*, *Yl *= *Yarrowia lipolytica*, *An *= *Aspergillus niger*, *Sp *= *Schizosaccharomyces pombe*, *Um *= *Ustilago maydis*.

The WASP proteins are characterised by an N-terminal WH1 domain followed by a basic domain (containing many lysins and arginines), a CRIB motif, a domain that we suggest to name WAID domain (WASP autoinhibitory domain), a unique region consisting of many proline residues (PPR, poly-proline region) and a VCA domain (Figure 1). In most of the previous descriptions of WASP proteins, the CRIB motif and the WAID domain have been described together as the GBD domain (GTPase binding domain). Here, we suggest regarding the CRIB motif and the WAID domain as distinct domains because they are structurally separated [14], they perform different functions, and they are differentially included into WASP and WAWH proteins. For example, while the fungi of the Chytridiomycota, the Blastocladiomycota, the Mucoromycotina, and the Basidiomycota branches contain both the CRIB and the WAID domain, the ascomycotes, the Schizosaccharomycota, and some of the yeast species only encode the WAID domain (Figure 1). Most yeast species sequenced so far lost both the CRIB and the WAID domain (Figure 1).

Several species encode multiple WASP homologs. To reveal subfamily relationships fragmented sequences and the unique regions were omitted from the multiple sequence alignment that was used to generate the phylogenetic tree (Supplementary Information). The tree shows that there have been many species-specific WASP duplications (e.g. in *Trichomonas vaginalis*, *Dictyostelium fasciculatum*, *Trichoplax adhaerens*, *Saccoglossus kowalevski*) and multiplications (e.g. in *Naegleria gruberi*, *Acytostelium subglobosum*, *Acanthamoeba castellanii*) but also two clade-specific duplications, one of which happened in the ancestor of the Mucoromycotina, and one at the onset of the vertebrates resulting in WASP and N-WASP. The duplication and subsequent subfunctionalization of the WASP homologs at the origin of the vertebrates is most probably the result of the two whole genome duplications (2R) that happened shortly after the emergence of the vertebrates [27]. WASP (but not N-WASP) has been lost in birds, and duplicates of both WASP and N-WASP are found in fish genomes. The additional duplications in fish genomes are probably the result of another whole genome duplication that happened at the origin of the Actinopterygii branch [28]. Some of the fish encode a further WASP homolog that is much shorter than the other WASP types and misses the basic, the central and the acidic domain (Figure 1, *Orn *S-WASP). Therefore, we suggest naming these WASP homologs S-WASP (short WASP). Similarly, some of the sequenced Basidiomycotes encode one or two further WASP homologs that do not encode a poly-proline rich region and a VCA domain. Although these short WASPs in fish and Basidiomycotes do not encode CA and VCA domains, respectively, that are the basis of ARP2/3-complex activation and therefore a requirement of WASP protein family membership, we would suggest to also group these proteins to the WASP family because of the strong sequence homology to the WH1 and CRIB/WAID domains of the long WASPs. Thus, they were most likely derived from WASP duplications followed by domain loss events. *Trichomonas vaginalis *encodes two special WASP homologs that have two (*Tv*WASP_B) and three (*Tv*WASP_A) tandem repeats of the poly-proline rich region and the VC domain but that miss the C-terminal acidic A domain (Figure 1). In comparison to a previous analysis [22] we were able to identify more WASP homologs even in species, which had been analysed before, and to complete sequences for which domains had been missing before. First, we also identified WASP homologs in *Trichomonas vaginalis *and the Haptophyte species *Emiliania huxleyi*. However, we found only one WASP homolog in *Caenorhabditis elegans *and all other nematode species. Second, there are invertebrate WASP homologs that do contain only one WH2 domain, like all Acoelomata WASPs sequenced so far, the tunicate *Oikopleura dioica *WASP, and one of the *Trichoplax adhaerens *homologs, *Tia*WASP_B. Our extensive analysis also allowed us to identify species outside the metazoa that do encode WASP homologs containing two WH2 domains like *Sphaeroforma arctica*, which belongs to the Fungi/Metazoa incertae sedis branch.

### Classification and analysis of the WAVE protein family

**Figure 2:**
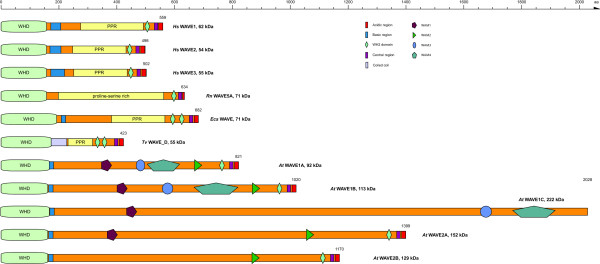
**Domain organisation of representative WAVE proteins**. A colour key to the domain names and symbols is given on the right except for the WHD domain (WAVE homology domain) and the poly-proline region (PPR) that are coloured in light green and yellow, respectively. Species abbreviations: *Hs *= *Homo sapiens*, *Ecs *= *Ectocarpus siliculosus*, *Tv *= *Trichomonas vaginalis*, *At *= *Arabidopsis thaliana*.

**Figure 3:**
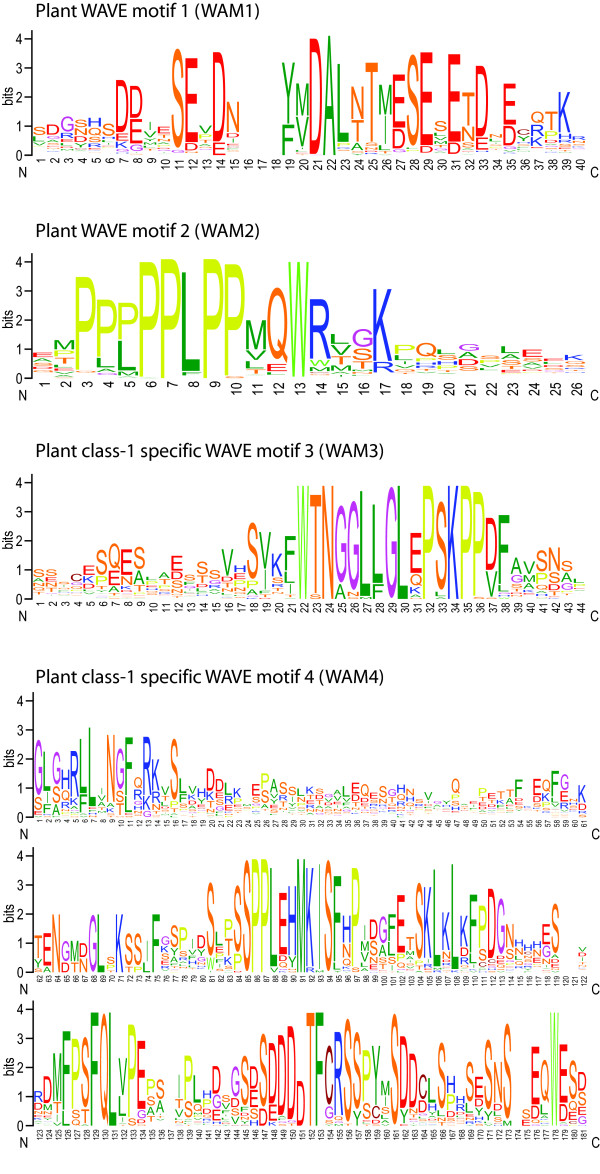
**Conserved sequence motifs and domains in plant WAVE proteins**. The sequence logos illustrate the sequence conservation within the multiple sequence alignments of three sequence motifs (WAM1, WAM2, and WAM3) and a domain (WAM4) that are specific to plant WAVE proteins. Plant WAVE proteins group into three different classes. WAM1 is present in almost all plant WAVE homologs, WAM2 has been introduced into plant WAVEs after the separation of the moss species, and WAM3 and WAM4 are unique to class-1 plant WAVEs.

In general, WAVE protein homologs consist of an N-terminal WHD domain followed by a basic region, a poly-proline region, and a C-terminal VCA domain (Figure 2). A few of the identified WAVE homologs are notable exceptions: 1) WAVE from the stramenopiles *Ectocarpus siliculosus *encodes two WH2 (V) domains (Figure 2). 2) Three of the eleven WAVE homologs of *Trichomonas vaginalis *encode short coiled-coils instead of the basic regions and also two WH2 domains. 3) The plant WAVE homologs do not contain poly-proline regions. Here, we could identify for the first time some plant-specific sequence motifs in the region between the basic and the VCA domain that we suggest to name WAM 1-4 (WAVE motif, Figure 3). Plant WAVE motif 1 (WAM1) is common to all plant WAVE homologs except the class-3 plant WAVE's, the *Arabidopsis *WAVE2B homologs, and the *Brassica rapa *class-2 WAVE. Thus, it is the most ancient of the plant WAVE motifs. The WAVE homologs that do not contain the WAM1 motif anymore seem to have lost it class- and species-specific. The WAM2 motif is characterised by a number of proline residues separated by leucine residues and followed by a glutamine-tryptophane-arginine triplett (Figure 3). This motif is present in all plant WAVE homologs except the *Physcomitrella patens *WAVE and *Arabidopsis *WAVE1C homologs. The motif has therefore been introduced in WAVE proteins after the separation of the moss species. Two further motifs were found in all class-1 WAVE homologs and are class-1 specific. WAM3 is a highly conserved motif while the WAM4 with a length of about 180 residues has the size of a domain (Figure 3).

**Figure 4:**
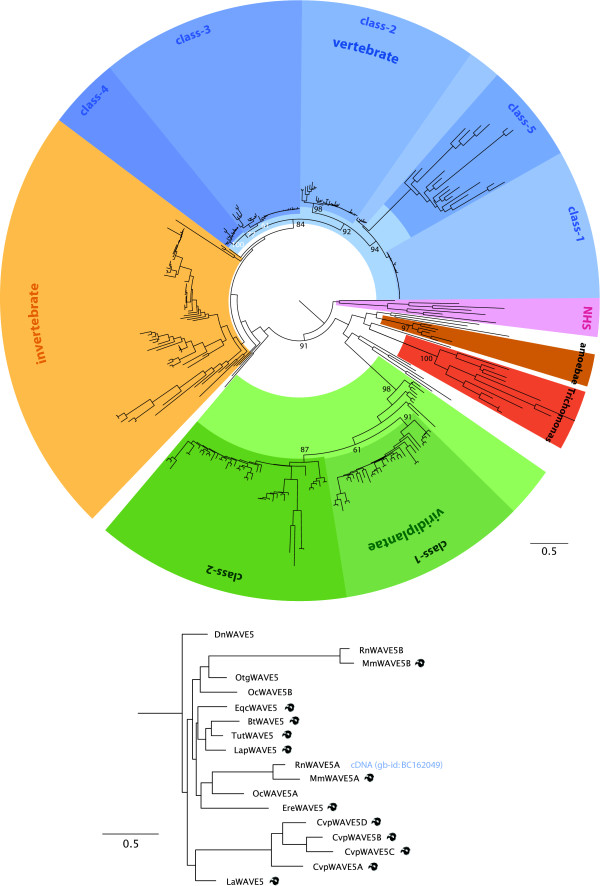
**Phylogenetic tree of the WAVE proteins**. The phylogenetic tree of the WAVE protein family was calculated from the multiple sequence alignment of the concatenated WHD and VCA domains using RAxML. For lack of space bootstrap support values are only given for major branches and indicated in percent. The WHD domains of a few NHS proteins have been used as outgroup. The unrooted tree was drawn with FigTree and branches were coloured according to class and taxonomic distributions. For the extended tree see Additional file 1.

Because many species encode more than one WAVE homolog we sub-grouped the WAVE proteins based on a phylogenetic tree calculated from concatenated multiple sequence alignments of the WHD and VCA domains (Figure 4). The plant WAVE homologs clearly group into two branches that we termed class-1 and class-2. Several plants contain further WAVE duplications, which are, so far, species-specific. Vertebrates contain WAVE homologs from up to five classes: A) The three well-known classes to which the human WAVEs belong. B) A fourth class that comprises WAVE homologs from all major vertebrate branches but mammalian WAVEs from only *Monodelphis domestica *and *Ornithorhynchus anantinus*. C) A fifth mammalian specific class that contains mainly pseudogenes but some potentially coding transcripts (Figure 4). Most mammals have already lost this class, like the primates, the dog, the panda bear, the bats, while others have up to six duplicates (*Cavia porrcellus*). Most of the members are potential pseudogenes because they are encoded by single exons, contain in-frame stop codons and frame-shifts, and sometimes miss stop-codons and starting methionines. Thus, there are more genetic disablements than can be attributed to sequencing and assembly errors. The class-5 WAVE homologs of *Rattus norvegicus*, *Oryctolagus cuniculus*, *Otolemur garnetii*, and *Dasypus novemcinctus *do not contain reading-frame interrupting mutations and might therefore present potentially expressed transcripts although they are all encoded by a single exon. The rat WAVE5A is the only class-5 WAVE supported by cDNA data. The mouse paralog WAVE5A, however, contains an in-frame stop codon.

### Classification and analysis of the WASH protein family

**Figure 5:**
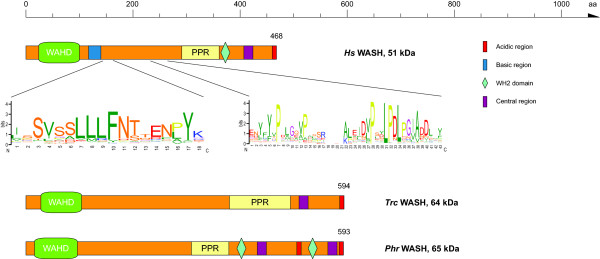
**Domain organisation of representative WASH proteins**. A colour key to the domain names and symbols is given on the right except for the WAHD domain (WASH homology domain) and the poly-proline region (PPR) that are coloured in light green and yellow, respectively. The sequence logos illustrate the sequence conservation within the multiple sequence alignments of two characteristic motifs shared by all WASH protein family members. Species abbreviations: *Hs *= *Homo sapiens*, *Trc *= *Trypanosoma cruzei*, *Phr *= *Phytophthora ramorum*.

Vertebrates and almost all other species analysed contain just one single functional WASH homolog as compared to the multiple homologs of WASP and WAVE proteins. The only exceptions so far are the amoebae *Dictyostelium fasciculatum *and *Acytostelium subglobosum*, and the Rhizaria *Bigelowiella natans *that encode two WASH homologs as result of species-specific gene duplications. In addition, several mammals, and especially primates, contain one or many WASH pseudogenes that are characterised by consisting of non-functional single exons because of frame-shifts, in-frame stop-codons, missing terminal stop-codons, and missing conserved sequence [19]. These pseudogenes are located in subtelomeric regions that are hotspots for rearrangements and interchromosomal duplications. It is unlikely but could be possible that some of these pseudogenes are in fact functional genes because of the general sequencing problems of these regions. WASH proteins contain a family-specific N-terminal domain that has been call WAHD domain (WASH homology domain, [4]) in analogy to the WH1 and WHD domains in WASP and WAVE, respectively. A short poly-proline rich region and a C-terminal VCA domain follow the WAHD domain (Figure 5). In contrast to the WASP and WAVE proteins the long region between the WAHD domain and the poly-proline region is not unique to species and taxa but contains several short motifs that are present in all WASH proteins (Figure 5). All WASH proteins have this general domain organisation except the *Trypanosoma species *WASH proteins, which miss the WH2 domain, and the Stramenopiles WASH proteins that encode two VCA domains arranged in tandem (Figure 5). Through the use of our manually curated sequence dataset the WASH homolog of *Phytophthora sojae *could be annotated correctly. While WASH and WAVE proteins are easily distinguished for most species, the WAHD domain of the *Phytophthora *species is the most divergent of all known WAHD domains. However, the WASH proteins of the *Phytophthora *species do encode the other characteristic motifs of the WASH family. Those features are clearly distinct from the WAVE homolog found in another sequenced Stramenopiles species, *Ectocarpus siliculosus*. Through manual inspection of the genomic DNA sequences together with manually maintaining the multiple sequence alignment we have been able to correct many automatic gene predictions and to greatly improve on initial bioinformatics studies [22]. Thus, we identified CA and VCA domains in the WASH homologs of kinetoplastid species (*Trypanosoma *and *Leishmania *species, Figure 5). In addition, the plant species *Physcomitrella patens *and *Selaginella moellendorfii *have a regular C-terminal acidic (A) domain.

### Classification and analysis of the WHAMM protein family

**Figure 6:**
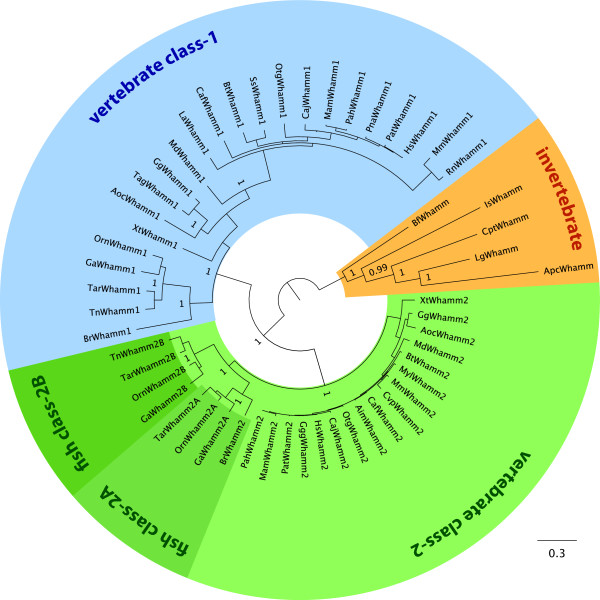
**Phylogenetic tree of the WHAMM proteins**. The phylogenetic tree of the WHAMM protein family was calculated with MrBayes from the multiple sequence alignment of the full-length WHAMM sequences excluding the low-complexity insertion of the WMD domain. Posterior probabilities are only given for some major branches because for lack of space. The unrooted tree was drawn with FigTree and branches were coloured according to class and taxonomic distributions. For the extended tree including all posterior probability values see Additional file 1.

The first member of this protein family, which has been shown to function as an actin nucleation promoting factor, has been named WHAMM [20]. Subsequently, a close homolog has been named JMY [21], and, most recently, invertebrate homologs have been identified and named WHAMY [22]. A phylogenetic tree of the available homologs shows that they all belong to the same protein family (Figure 6). The grouping is similar to that of other metazoan protein families showing single members for the invertebrates and duplicates for the vertebrates that are the result of the 2R genome duplication at the origin of the vertebrates. Another genome duplication followed the divergence of the fish resulting in some more duplicates in fish genomes. Thus, all members of this protein family should get the same name, which should be WHAMM because this was the first member identified to function as NPF.

**Figure 7:**
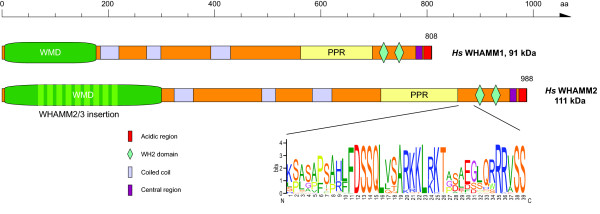
**Domain organisation of representative WHAMM proteins**. A colour key to the domain names and symbols is given on the right except for the WMD domain and the poly-proline region (PPR) that are coloured in dark green and yellow, respectively. The class-2 and class-3 WHAMM homologs encode insertions in the WMD domain. For *Hs*WHAMM2 the insertion is symbolized by light-green bars. The sequence logo illustrates the sequence conservation within the multiple sequence alignments of the previously supposed third WH2 domain in class-2 WHAMM homologs. However, the sequence is not in accordance with the consensus WH2 sequence motif. Species abbreviation: *Hs *= *Homo sapiens*.

Previously, the N-terminal domain of WHAMM has been defined differently, sometimes marking the first 50 residues at the N-terminus [21], sometimes comprising the first 170 residues of mammalian WHAMM1 [20,29]. In the first case, the N-terminal domain ends before a long (about 120 aa) low-complexity region in WHAMM2 homologs, while in the other case the N-terminal domain is extended subsequent to this low-complexity region by another 120 residues that are conserved between all WHAMM homologs. Because all invertebrate WHAMM homologs also do not contain the low-complexity region, it is most likely that it has been inserted into the N-terminal domain of WHAMM2 after the vertebrate WHAMM duplication happened. Therefore, we support the definition of the longer N-terminal domain that has been called WMD (WHAMM membrane interaction domain, Figure 7, [30]).

We have also found a WHAMM homolog in the Hydrozoa species *Clytia hemisphaerica*, which is outside Bilaterians and thus extends a previously proposed origin of WHAMM homologs [22]. The WHAMM homolog of *Strongylocentrotus purpuratus *has been proposed to contain a tandem N-terminal WMD and coiled-coil region [22], but this is most likely an artefact from the genome assembly. In fact, the duplicated part (WMD domain and coiled-coil region) is surrounded by stretches of 'N' bases and has most probably misleadingly been placed inside the supercontig containing the WHAMM gene during the genome assembly process. The *Strongylocentrotus *genome is one of the worst assemblies available because of the high polymorphism between the two alleles. Therefore, not even the well-conserved myosin family proteins could unambiguously be annotated [25].

It has been reported initially [21] and subsequently adopted in recent reviews [4,30] that class-2 WHAMM homologs (JMY) contain three WH2 domains. The region comprising the supposed first WH2 domain is highly conserved in all vertebrates (Figure 7). However, this region does not show homology to the WH2 regions of the WASP family proteins (see also below) that are characterised by a highly conserved pattern ([VILM]-[LM]-[ASED]-[ASDEQ]-[IL]-[KRQ]-x10-[L]-[KR]-[KR]-[VTA]). The prediction of a third WH2 domain in class-2 WHAMM homologs is thus most likely an artefact, or the respective region would comprise an extremely unusual WH2 domain. There is no experimental data available supporting this motif being a true WH2 domain.

### Identification and domain organisation of the WAWH and WAML families

**Figure 8:**
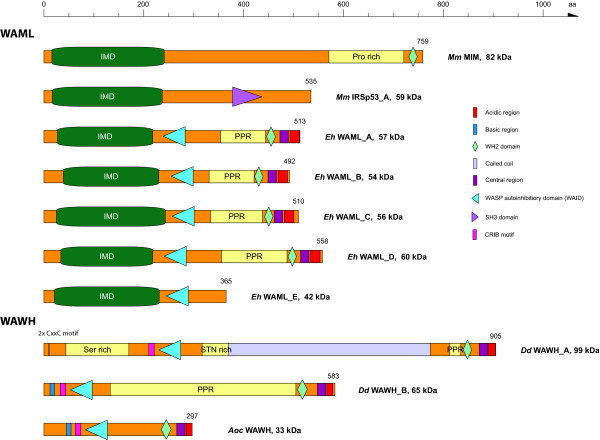
**Domain organisation of representative WAWH and WAML proteins**. A colour key to the domain names and symbols is given on the right except for the IMD domain and the poly-proline region (PPR) that are coloured in dark green and yellow, respectively. Species abbreviations: *Mm *= *Mus musculus*, *Eh *= *Entamoeba histolytica*, *Dd *= *Dictyostelium discoideum*, *Aoc *= *Anolis carolinensis*.

While we extensively searched the eukaryotic genomes for WASP homologs we observed several hits for the GBD and VCA domains in amoebozoan genomes. The true WASP homologs could easily be identified and assembled because of homology hits for the WH1 domain. When we assembled the first of the hits in *Dictyostelium discoideum*, for which only homology to the GBD and VCA domains was indicated, we were long searching for potential WH1 domains upstream of the GBD domains, expecting even very low sequence similarity. However, we were not able to identify any WH1 domain, and when we analysed further amoebozoan genomes it became clear that we have identified a new type of the WASP protein family, that we suggest to call WAWH (WASP without WH1 domain). The WAWH proteins have a similar domain organisation as the WASP proteins, except that they miss the N-terminal WH1 domain (Figure 8). Three different WAWH sub-types have been found: A) Type-I that has the same domain organisation as WASP except for the WH1 domain that is missing. B) Type-II that does not have the basic region N-terminal to the GBD domain but has two conserved CxxC motifs at the N-terminus followed by a serine-rich region, and a long coiled-coil region between the GBD domain and the PPR region. C) Type-III WAWHs have a highly charged region (about 90% of the sequence consists of glutamate, arginine, and lysine) of about 800 residues between the GBD domain and the PPR region. While all amoebae encode one or more of the type-I and type-II WAWHs, the type-III WAWH has so far only been found in *Entamoeba *species. WAWH homologs have also been identified in the Apusozoa *Thecamonas trahens *and the lizard *Anolis carolinensis*.

Because all amoeba genomes contain at least one WASP gene we were searching for WASP homologs in *Entamoebae *for a long time. Low-scoring hits were only obtained for the WH2 domain, and because this domain is very short (*Entamoebae *genes rarely contain introns so that we would have expected longer hits if there would be homology to neighbouring regions, which we would expect for WASP homologs) and also present in many other proteins we did not analyse these hits further in the first instance. As soon as further amoebozoan genomes became available we always searched the *Entamoeba *genomes again for WASP homologs. As we started to inspect the short hits for the WH2 domains further, the corresponding assembled genes revealed a similar domain organisation as WASP proteins starting with the WAID domain (Figure 8). Surprisingly, five to seven proteins with a similar domain organisation have been found in the three sequenced *Entamoeba *genomes. In contrast to WASP proteins, these proteins each encode an IMD domain (IRSp53/MIM homology domain) at the N-terminus and lack the CRIB motif. IMD domains are found in proteins of the IRSp53 (insulin receptor tyrosine kinase substrate p53) and proteins of the MIM (missing-in-metastasis) family, for example. The MIM family proteins additionally contain proline-rich regions and a C-terminal WH2 domain (in one of the alternatively spliced transcripts). We therefore suggest calling these new types of WASP family proteins WAML (WASP and MIM like). One subclass of the WAML proteins comprises a short version that misses the PPR and VCA region.

### Comparison of the WH2 and central domains

**Figure 9:**
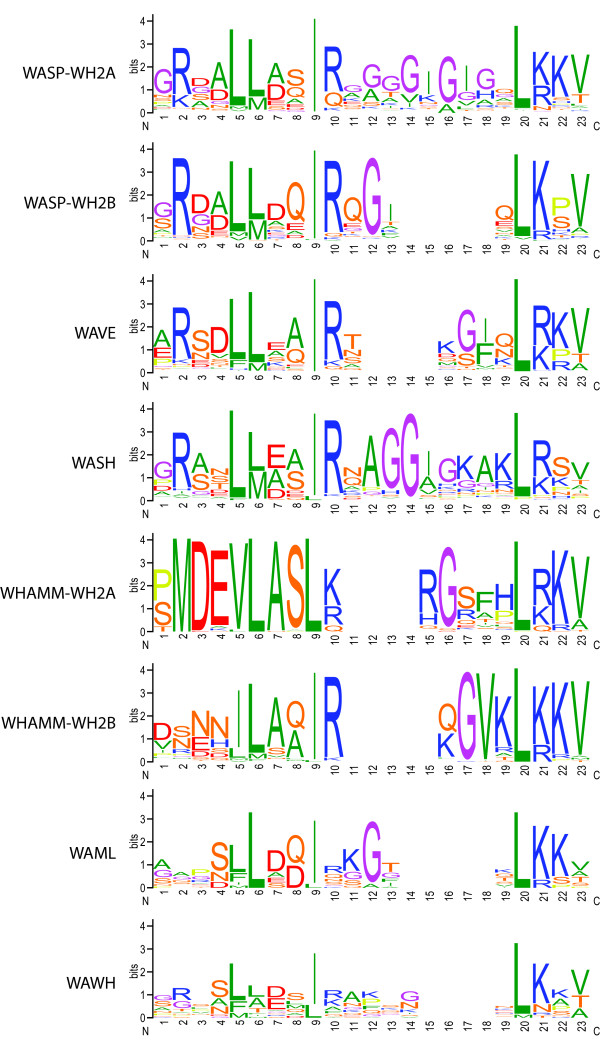
**Sequence conservation of the WH2 domains of WASP family proteins**. The sequence logos illustrate the sequence conservation within the multiple sequence alignments of the WH2 domains. Several of the WASP proteins as well as the vertebrate WHAMM homologs encode two WH2 domains in tandem that are shown separately here. The linker between the N-terminal helix part of the WH2 domain and the C-terminal LKKT motif are of variable length. To align all WH2 domains to each other, four to five gaps have been introduced into the linker of all but the WASP-WH2A and WASH alignments. The sequence logos are based on the following number of sequences: WASP-WH2A = 343; WASP-WH2B = 110; WAVE = 321; WASH = 141; WHAMM-WH2A = 63; WHAMM-WH2B = 69; WAML = 14; WAWH = 19.

The WH2 domain is a short sequence motif of 17-27 residues and binds to actin at the barbed-end [31-33]. The first ten amino acids of the WH2 domain form a short alpha-helix that binds to the hydrophobic cleft between subdomains 1 and 3 of actin. A linker of variable length follows the helix. After the linker a strongly conserved LKKT(V) motif anchors the C-terminal part of the WH2 domain into a hydrophobic pocket (via the leucine) while directing the C-terminal end of the WH2 domain towards the pointed-end of actin. The WASP family proteins contain well-conserved WH2 domains (Figure 9). Based on crystal structures of WH2 domains in complex with actin the N-terminal helix is characterised by two highly conserved large hydrophobic residues (VILM) followed by two residues, which might be small (AS), polar (SQ) or negatively charged (DE), and another completely conserved large hydrophobic residue (I or L). The hydrophobic residues bind into the hydrophobic cleft of actin's barbed-end. The linker almost always contains a hydrophobic residue (VIF) that binds into a hydrophobic pocket on the actin surface. Based on the 1080 WH2 domain sequences of the WASP family proteins the following pattern can be identified: ([VILM]-[LM]-[ASED]-[ASDEQ]-[IL]-[KRQ]-x10-[L]-[KR]-[KR]-[VTA]). The proposed third WH2 domain of class-2 WHAMM homologs does not show homology to the N-terminal helix part of the motif and should therefore not be able to form a similar structure. The WH2 domains are highly conserved throughout all WASP family proteins (Figure 9) containing linker of four to five residues. WASH protein WH2 domains and fungi WASP-WH2 domains (included in the WASP-WH2A sequence logo in Figure 9) contain longer linker of ten and seven to eight residues, respectively. The highest conserved residues of all WH2 domains are the three hydrophobic amino acids of the N-terminal alpha-helix and the LKKV motif. The WHAMM-WH2A sequence logo comprises only vertebrate sequences and is therefore highly conserved over the entire length of 19 residues of the domain.

**Figure 10:**
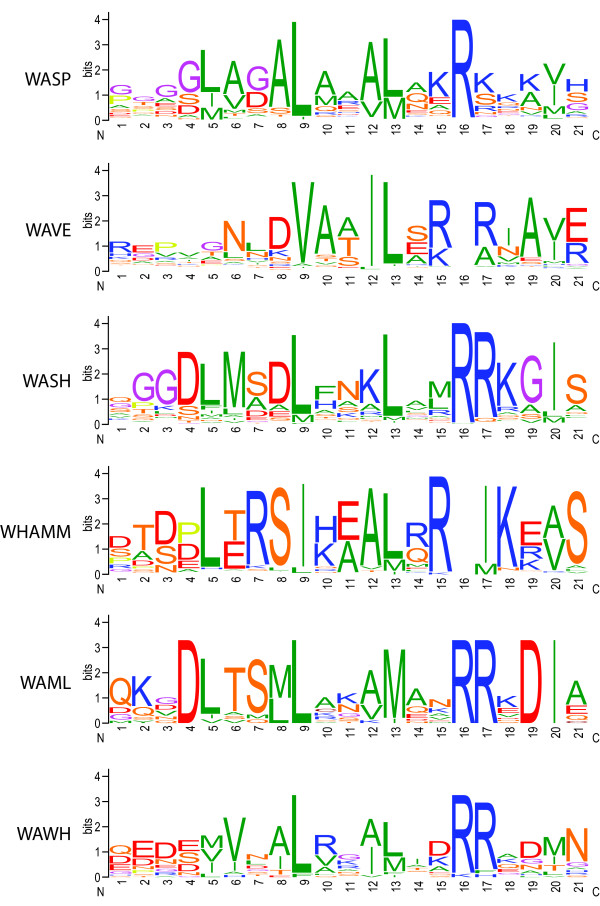
**Sequence conservation of the C domains of WASP family proteins**. The sequence logos illustrate the sequence conservation within the multiple sequence alignments of the C domains. The sequence logos are based on the following number of sequences: WASP = 349; WAVE = 321; WASH = 154; WHAMM = 69; WAML = 14; WAWH = 19.

In contrast to the WH2 domains, the C domains of the VCA region have WASP sub-family specific patterns (Figure 10). The general pattern of the C domain can be depicted as follows: The center of the motif is characterised by three large hydrophobic residues (VILM) that are each separated by three amino acids. A doublet or triplet of arginine or lysine residues follows. The C domain is terminated by another large hydrophobic residue (VILMY). The hydrophobic residues are almost completely conserved in each subfamily, and the specific combinations could be used as marker. The C domains show stronger sequence conservation than the WH2 domains.

The acidic C-termini of the WASP family proteins are thought to mediate ARP2/3 binding. Most acidic domains contain a tryptophan residue as last coding residues or close to the terminus. While phenylalanines instead of the tryptophans have been reported to be unique to invertebrate WHAMM homologs [22] we have identified WASP homologs, which contain F's instead of W's (e.g. in Acoelomata), WASH homologs without any hydrophobic residues in the A domain (e.g. *Leishmania *WASH proteins), and many WASP and WAVE homologs that contain both a tryptophan and a phenylalanine/tyrosine, both separated by variable numbers of acidic residues. Thus, it remains an open question whether a tryptophan or just an aromatic residue is needed for proper function, and whether there is a difference in encoding only a tryptophan or an additional phenylalanine/tyrosine.

### Alternative splice forms of WASP family proteins

Surprisingly, alternative splice forms of WASP family proteins have not been reported yet except for a neural-specific splicing of WASP through that a truncated WASP protein is generated lacking exon-2 [34]. Exon-2 encodes the N-terminal five ß-strands of the seven ß-strands of the WH1 domain [35] and it seems impossible that a functional and folded WH1 domain could be formed without the exon-2 encoded residues. We have extensively searched all cDNA/EST databases available of all species for clones encoding the reported exon-2 deletion but have not found any. Interestingly, none of the four to nine isoforms of the three mammalian homer proteins, that also contain an N-terminal WH1 domain, encodes a sequence in which part of the WH1 domain is missing [36]. The exon-2 deletion might thus be a very limited event, so that it has not yet been included in cDNA/EST libraries (although dozens of cDNA/EST clones from many species exist for the full-length WH1 domain), or it might be an experimental artefact. The available data argues for the latter.

**Figure 11:**
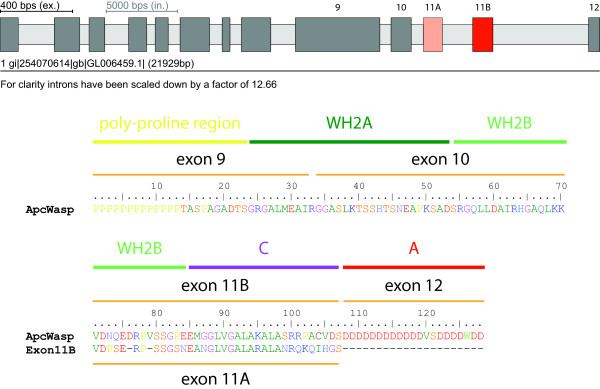
**Gene structure of the alternatively spliced WASP of *Aplysia californica***. The cartoons outline the gene structure of the alternatively spliced WASP gene from *Aplysia californica*, *Apc*WASP. The *Apc*WASP gene contains a cluster of two mutually exclusive spliced exons, exon11A and exon11B, that span the C-terminal part of the second WH2 domain and the C domain. Dark grey bars and light grey bars mark exons and introns, respectively, and alternative exons are coloured.

We have searched all available databases for cDNA/EST clones representing different splice forms of the WASP family proteins. We were not able to identify any alternatively spliced exons except for a mutually exclusive spliced exon of the *Aplysia californica *WASP gene (*Apc*WASP; Figure 11). The cluster of these mutually exclusive spliced exons encodes alternative versions of part of the second WH2 domain and the C domain, which is responsible for the autoinhibition of the WASP proteins. The autoinihibition is released through binding of activated GTPases, and one possible function of the two different versions of the C domain of *Apc*WASP might be to form modulated autoinhibited complexes that will be activated by different GTPases. But this is pure speculation and experimental evidence is needed to reveal a precise mechanism. Based on these findings we have searched all genomes of the closest related sequenced species (e.g. *Lottia gigantea*, *Capitalla teleta*, *Helobdella robusta*) for mutually exclusive spliced exons but could not identify any.

This situation is in sharp contrast to other proteins sharing WASP domains like the NHS proteins or the homer proteins. The Nance-Horan syndrome protein has an N-terminal WHD domain like the WAVE proteins, is alternatively spliced, and encodes at least five isoforms [37]. The three mammalian homer family proteins have N-terminal WH1 domains (EVH1 domain) like the Ena/VASP and the WASP proteins and encode four to nine different isoforms [36].

## Discussion

Initially, the WASP family of proteins consisted of the WASP proteins and the WAVE proteins [7,38]. Both share the ability to catalyze actin filament branching via activation of the ARP2/3 complex but are localized and regulated differently. The shared function is encoded in a similar subsequent set of domains located at the C-termini. A poly-proline or proline-rich region is followed by a WH2 domain, a central domain, and a C-terminal acidic domain that together are called the VCA domain. This set of domains has very recently been identified in further proteins, WASH [19], WHAMM [20], and JMY [21] that have also been shown to activate the ARP2/3 complex. The common characteristic of all WASP family proteins is thus the VCA domain following a proline rich region. The aforementioned proteins are regarded as subfamilies, and newly identified proteins containing a VCA domain but different N-terminal domains should be regarded as new subfamilies. The N-terminal domains defining the subfamilies are also present in other proteins (for example the WH1 domain is present in Ena/VASP proteins, the WHD domain in NHS proteins, and the IMD domain in IRSp53 and missing-in-metastasis proteins) and therefore most WASP family proteins also belong to other protein families.

The analysis of most eukaryotic sequenced genomes available allowed us to reconstruct the evolution of the WASP family members in the different branches of the eukaryotic tree and to identify and characterise new subfamilies. It is very important to analyse as many genomes as possible because even closely related species might contain different sets of the WASP subfamilies and subfamily members. For example, the Entamoeba species *Entamoeba histolytica *and *Entamoeba dispar *both contain one WASH protein, and three WAWH and five WAML homologs, while the close relative *Entamoeba invadens *has lost WASH but instead encodes three WAWH and seven WAML homologs. Another example represents the green algae species sequenced by today. For the *Ostreococcus *species a WASH homolog could be identified, while WASP family homologs could not be identified in *Volvox carteri *and *Chlamydomonas reinhardtii *although both have ARP2/3, and the *Micromonas *and *Chlorella *species do not have the ARP2/3 complex anymore and thus do not encode any WASP family protein. Therefore, it is not sufficient to only analyse a few sample genomes of each of the main eukaryotic kingdoms as it has been done in previous analyses [22,39]. Of course, sequencing of further genomes might also lead to a revision of the conclusions drawn below.

For species for which we were not able to identify any WASP family protein homolog we searched for all actin and actin-related protein genes. If these species also did not contain members of the ARP2 and ARP3 subfamilies the absence of ARP2/3-complex activating proteins was confirmed. However, we identified ARP2 and ARP3 in some species for which we were not able to find WASP family protein members yet. Based on the comparison of about 2,200 homologs of actin-related proteins the identification of ARP2 and ARP3 homologs is straightforward (data not shown). This is in contrast to the search for homologs of the other four members of the pentameric WAVE complex. Each of the other proteins of the WAVE complex is part of a protein family whose members are also incorporated into the WASH complex [23]. In addition, the WAVE complex subunits also interact with the NHS (Nance-Horan syndrome) proteins [37]. The Nance-Horan syndrome is, like the Wiskott-Aldrich syndrome, linked to the X-chromosome. The protein causing the syndrome, the NHS protein, contains an N-terminal WHD domain like the WAVE proteins [37,40]. Three NHS family members exist in humans, NHS, NHSL1, and NHSL2, and it could be shown that NHS also regulates actin remodelling and cell morphology [37]. Interestingly, the WHD domain of NHS interacts with all three members of the Abi protein family, Nap1, Sra1, and HSPC300 [37], as has been shown for the mammalian WAVE proteins [17,18,41]. Thus, if we had wanted to confirm the presence or absence of WAVE or WASH proteins in certain species by searching for the presence of the other members of the WAVE and WASH complexes we would have had to perform an exhaustive analysis of all members of the complexes as well as the NHS proteins. This is, however, out of the scope of this analysis, and we therefore rely on our experience in WASP protein family identification to approve their presence or absence.

**Figure 12:**
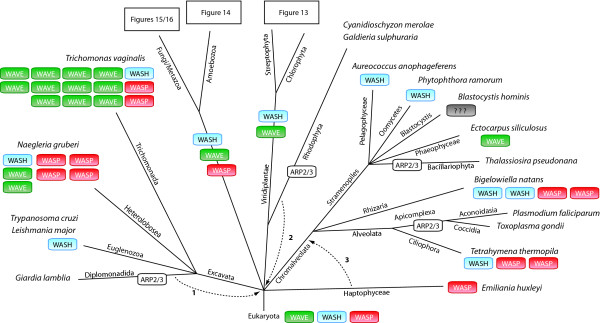
**Evolution of the WASP family proteins with respect to the species evolution**. Schematic representation of the most widely accepted eukaryotic tree of life. Branch lengths are arbitrary. The WASP family protein inventories of certain taxa and specific species have been plotted to the tree with WASP subfamilies given in colour-coded boxes. White boxes in certain lineages or species indicate the loss of the ARP2/3 complex. *Blastocystis hominis *contains the ARP2/3 complex and is thus expected to contain an activator protein although none of the WASP family members could be identified. The numbers on the arrows refer to alternative placing of the respective taxa: **1**: The monophyly of the Diplomonadida (instead of grouping them to the superkingdom Excavata) is supported by [54]. **2**: The monophyly of the Rhodophyta is supported by [45,48]. **3**: Grouping the Haptophyceae to the Chromalveolata is supported by [45-47].

Having identified all WASP family homologs in all sequenced organisms, the presence or absence of the various homologs in the respective species can now be plotted on the tree of the eukaryotes (Figure 12). As basis for the tree of the eukaryotes we choose the taxonomy as available at NCBI [42] with many modifications based on recently published phylogenetic analyses. Especially the grouping of taxa that emerged close to the origin of the eukaryotes remains highly debated. According to most of the recent phylogenetic analyses, the Alveolata, Stramenopiles, and Rhizaria form the superfamily SAR [43,44]. The placement of the Haptophyceae to the SAR is still highly debated and there are several analyses in favour [45-47] as well as in contrast [43,44,48-50] to this grouping. Also, some analyses group the red algae from the Rhodophyta branch to the Viridiplantae [44,50,51] and others support their independence [45,48]. The phylogeny of the supergroup Excavata is the least understood because only a few species of this branch have been completely sequenced so far. While the grouping of the Heterolobosea, Trichomonada, and Eugelenozoa into the Excavata is found in most analyses, the grouping of the Diplomonadida as separate phyllum or as part of the Excavata is still highly debated although most recent analyses support the Excavata superkingdom [43,48,49]. Also, we placed the nematodes as sister group to the arthropods forming together the Ecdysozoa clade [52,53]. The other sequenced Protostomia species form the Lophotrochozoa branch [52].

Our analysis shows that the most ancient eukaryote must have contained a WASP, a WAVE, and a WASH homolog, because homologs of these subfamilies have been found in all major eukaryotic branches (Figure 12). Some species contain extended repertoires of WASP family proteins like *Trichomonas vaginalis *encoding eleven WAVE homologs. In contrast, the Euglenozoa contain only a WASH homolog, while the Stramenopiles encode either a single WASH or a single WAVE protein, and the Haptophyceae *Emiliania huxleyi *contains only a WASP homolog. The ARP2/3 complex, and thus WASP family proteins, is absent in Diplomonoda, Rhodophyta, Bacillariophyta, and Apicomplexa. The Stramenopiles *Blastocystis hominis *contains the ARP2/3 complex, but WASP family proteins could not be found in its genome. Here, proteins from class II NPFs might regulate the ARP2/3 complex.

**Figure 13:**
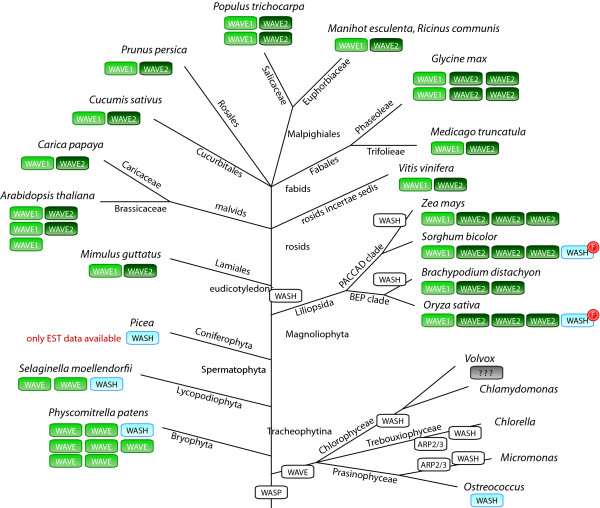
**Evolution of the WASP family proteins in algae and plants**. Schematic representation of the phylogenetic tree of the sequenced algae and plant species. Branch lengths are arbitrary. The WAVE and WASH protein inventories of the species have been plotted to the tree with WASH and WAVE families and classes given in colour-coded boxes. Loss of the ARP2/3 complex and WASP family proteins in certain lineages or species is indicated by white boxes. *Volvox carteri *and *Chlamydomonas reinhardtii *contain the ARP2/3 complex and are thus expected to contain an actin nucleation promoting protein although none of the WASP family members could be identified. The red "F" indicates that only a fragment of the WASH protein in *Sorghum *and *Oryza *is available. The genome assemblies of these species are of high quality and do not contain any gaps downstream of the WASH fragments. Because the found N-terminal fragments are highly similar to the respective regions of the WASH homologs of *Picea, Selaginella*, and *Physcomitrella *but homologous sequence to the rest of the WASH proteins could not be identified in the genome sequences these WASH proteins are most probably pseudogenes in the process of being disbanded.

WASP proteins have never been identified in plant genomes [39,55], and we were also not able to find any WASP homolog in plants (Figure 13). Thus, the ancestor of the plants must have lost the WASP homolog. However, we identified WASH homologs in green algae of the *Ostreococcus *species and some land plants that separated very early in the evolution of the viridiplantae, like the moss *Physcomitrella patens *and the fern *Selaginella moellendorfii*. Based on these WASH proteins we searched in all other available plant genomes for homologs. A full-length WASH homolog has only been identified in cDNA data of *Picea glauca *and fragments of very similar sequences to the *Picea *WASH in cDNA data of another Coniferophyta, *Pinus taeda*, and the Cycadophyta *Cycas rumphii*. N-terminal fragments of WASH have been identified in the genomes of the Liliopsida *Sorghum bicolour*, *Oryza *species, *Phoenix dactylifera*, and *Setaria italica*. These fragments comprise the N-terminal about 200 residues, and no further WASH sequence could be identified in spite of extensive manual inspection of the genomic DNA sequences. Because the C-termini of these fragments are not conserved and because homologous sequence to these fragments could not be identified in other Liliopsida genomes it is most probable that these fragments are pseudogenes in the process of being disbanded. The analysis also shows that WASH has been lost in the last common ancestor of the eudicotyledons branch. Although several good assemblies of genomes of green algae are available, WASH homologs (and the ARP2/3 complex) could only be identified in the *Ostreococcus *species (Figure 13). The *Chlorella *and *Micromonas *species do not encode ARP2 and ARP3 and it is thus not surprising that they also do not contain WASH. However, the Chlorophyceae species *Volvox carteri *and *Chlamydomonas reinhardtii *do contain ARP2 and ARP3 but WASH or WAVE homologs were not found. In these species the ARP2/3 complex must be activated by another type of WASP family protein that has not been recognized so far. In all genomes of land plants several WAVE homologs have been identified. While the WAVE homologs of *Physcomitrella *(containing a surprisingly high number of seven WAVEs) and *Selaginella *consistently group together in the phylogenetic tree of the WAVE proteins, the WAVE homologs of the Magnoliophyta clearly group into two major classes. Variants of these classes, like those in *Arabidopsis *and *Populus*, have been derived by species-specific duplications.

**Figure 14:**
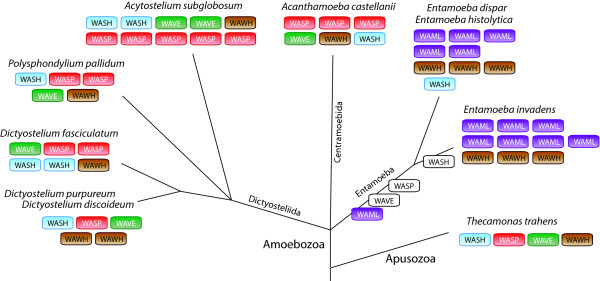
**Evolution of the WASP family proteins in amoebae and Apusozoa**. Schematic representation of the phylogenetic tree of the sequenced amoebae and Apusozoa species. Branch lengths are arbitrary. The WASP family protein inventories of the species have been plotted to the tree with WASP families given in colour-coded boxes. White boxes indicate losses of WASP family proteins in the Entamoeba lineage.

The amoebae have a rich repertoire of WASP family proteins (Figure 14). The Dictyosteliida and *Acanthamoeba castellanii *contain WASP, WAVE, and WASH homologs, and also homologs of the newly identified WAWH proteins. In addition, most amoebae species sequenced so far contain several species-specific gene duplications. In contrast, the Entamoeba species have lost WASP and WAVE, but instead encode five or more WAML homologs. WAML proteins contain an N-terminal IMD (IRSp53-MIM homology domain) domain, a WAID domain, a proline-rich region, and a C-terminal VCA domain. The VCA domain of WAML might activate the ARP2/3 complex in a similar way as the VCA domains of WASP and WAVE protein. Because the Rho-GTPase binding domain is missing in WAML there must be a different way to release the VCA domain from the intramolecular binding to the WAID domain. What might be the function of the IMD domain in WAML proteins? N-WASP is known to be essential for the formation of distinct cellular architectures such as endocytic vesicles, filopodia and podosomes/invadopodia [7]. These structures are formed by the action of recently identified classes of membrane-deforming proteins, which bind to phospholipids and deform membranes to curved surfaces [56,57]. These proteins group into three subfamilies containing either a BAR domain, an EFC/F-BAR domain, or an RCB/IMD domain [58]. The SH3 domains of these membrane-deforming proteins can interact with the WASP and WAVE proteins, recruit these to the membrane and thus shape unique membrane-cytoskeleton architectures. In addition, IMD domains but not BAR domains have also been shown to bind to and bundle actin filaments [32,59,60]. The WAML proteins might represent a fusion of the membrane-deforming function of the BAR/IMD proteins, the actin-bundling function of the IMD proteins, and the ARP2/3 activating function of the WASP/WAVE proteins. The high number of different WAML homologs might be necessary to specifically form the distinct cellular architectures. The WAWH homologs in Entamoeba could be responsible for ARP2/3 dependent processes not located at the cell surface.

**Figure 15:**
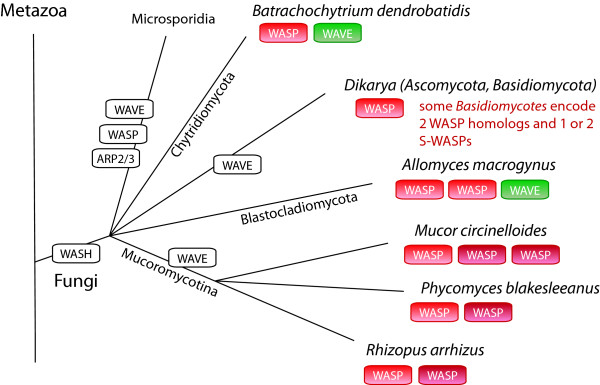
**Evolution of the WASP family proteins in fungi**. Schematic representation of the phylogenetic tree of representative fungi branches and species. Branch lengths are arbitrary. The WASP family protein inventories of certain branches and some specific species have been plotted to the tree with WASP families given in colour-coded boxes. White boxes indicate losses of the ARP2/3 complex and WASP family proteins in the respective lineages.

All fungi that have been sequenced so far have lost the WASH homolog (Figure 15). In addition, the Dikarya (containing the Ascomycotes and Basidiomycotes) and Mucoromycotina also lost the WAVE homolog. The ancestors of the Blastocladiomycota and Mucoromycotina have duplicated the WASP gene independently of each other. Some of the Basidiomycotes also encode an S-WASP (short WASP, ending shortly behind the WAID domain) suggesting that the S-WASP must have been invented very early in Basidiomycote evolution and subsequently been lost in most Basidiomycotes. The Microsporidia do not contain an ARP2/3 complex and thus do not encode any protein of the WASP family. The missing WASP protein family complexity in Ascomycotes and Basidiomycotes compared to the other fungi lineages, plants, amoebae, and metazoans might be compensated by other proteins. For example, coronins [61,62] and the class-1 myosins [63,64] contain Ascomycote- and Basidiomycote-specific additions of CA domains to their protein domain organisation through which they can bind and regulate the ARP2/3 complex.

**Figure 16:**
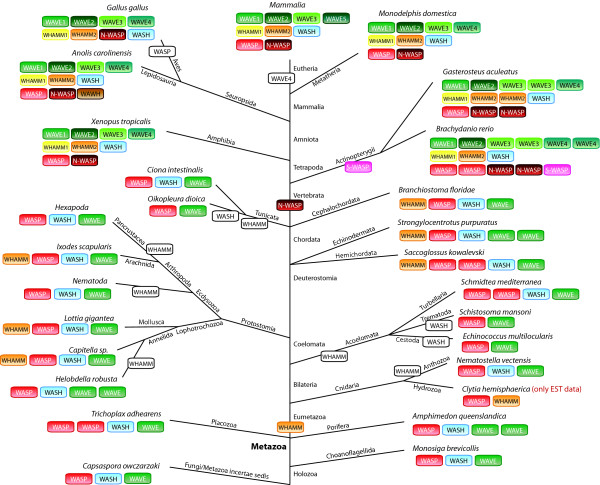
**Evolution of the WASP family proteins in metazoa**. Schematic representation of the phylogenetic tree of representative sequenced metazoan species. Branch lengths are arbitrary. The WASP family protein inventories of the species have been plotted to the tree with WASP families given in colour-coded boxes. White boxes indicate losses of WASP family proteins in the respective lineages and species.

Most of the invertebrate species analyzed here contain one of each of the WASP, WAVE, and WASH homologs (Figure 16). In a few cases, one of these proteins has been duplicated in a species-specific manner or in recently separated branches. Surprisingly, the WHAMM protein has been invented very early in metazoan evolution, at the onset of the eumetazoans, which is based on the identification of a WHAMM homolog in the Hydrozoa *Clytia hemisphaerica*. However, most of the invertebrate species sequenced so far have subsequently lost the WHAMM homolog. In addition, the Trematoda *Schistosoma mansoni *and all sequenced Cestoda have lost the WASH homolog. At the origin of the vertebrates two whole-genome duplication events happened [27] followed by another whole-genome duplication event in the Actinopterygii branch [28,65]. These duplications are, at least in part, represented in the WASP family protein inventory of the sequenced vertebrate species. WASH is the only WASP family protein that has not been duplicated, although some WASH pseudogenes could be identified in primate genomes. WASP has been duplicated resulting in one of each WASP and N-WASP in tetrapods, and several further duplications of both in fish. Some of the sequenced fish in addition contain a short WASP (S-WASP) that is characterised by a very short poly-proline region and the absence of the basic and the CA domains. Because of the absence of the CA domains these WASP homologs should not be able to bind and activate the ARP2/3 complex. Similar to WASP, the tetrapods encode two homologs of the WHAMM proteins, a class-1 and a class-2 WHAMM, and several fish contain further duplicates of the class-2 WHAMM proteins. Thus, during or shortly after the two whole-genome duplications at the onset of the vertebrates, the WASH duplicates and two of each of the WASP and WHAMM duplicates have been lost. This is in contrast to the WAVE proteins. The vertebrate WAVE homologs can be divided into four classes, of which the class-1 and class-2 WAVE proteins are more closely related to each other as are the class-3 and class-4 WAVE proteins than these two groups of classes are related. This could best be explained by two subsequent gene-duplications. The fish have further WAVE duplicates in some classes that are the result of their additional whole-genome duplication. The Eutheria have lost the class-4 WAVEs. Instead, many Eutheria encode one to seven further WAVE homologs, class-5 WAVEs. Most of them are, however, most likely pseudogenes because of frame-shifts, in-frame stop-codons, missing promoters and stop-codons, and because they would be encoded by a single exon in contrast to all other vertebrate WAVE homologs that are encoded by several exons. In addition, most species have completely lost this WAVE variant. The rat WAVE5A is the only class-5 WAVE supported by cDNA data. Interestingly, the lizard *Anolis carolinensis *also encodes a WAWH homolog based on its domain composition.

## Conclusions

We could show that most eukaryotes encode a rich repertoire of WASP family proteins to regulate the ARP2/3 dependent remodelling of the actin cytoskeleton. Surprisingly, alternative splice variants could be identified for only a single WASP homolog from the sea hare *Aplysia californica*. This is in strong contrast to other actin-binding proteins that have the same N-terminal domains like Ena/VASP (N-terminal WH1 domain), NHS (N-terminal WHD domain), and MIM (N-terminal IMD domain). Single transcripts are most probably a characteristic of the WASP family proteins because extensive cDNA data is already available. Given the recent identification of two new WASP protein subfamilies, WASH and WHAMM, and of the two new subfamilies described here, WAML and WAWH, and in the prospect of many upcoming genome sequences the WASP protein family is expected to grow further.

## Methods

### Identification and annotation of the WASP family genes

WASP family genes have been identified in TBLASTN searches of the completed or almost completed genomes of 529 organisms starting with the protein sequences of the human homologs. Species outside the metazoans that were expected to encode very divergent homologs were searched with many different homologs and every single hit showing homology to either the C-terminal domains (WH2, central, and acidic domains), the GBD domain, or the N-terminal domains was analysed in detail. During this search, the new WASP subfamilies WAWH and WAML were identified based on the homology of their GBD domains to those from WASP proteins. All hits were manually analysed at the genomic DNA level. The correct coding sequences were identified with the help of the multiple sequence alignment of the respective protein family. As the amount of sequences increased (especially the number of sequences of taxa with few representatives), many of the initially predicted sequences were reanalysed to correctly identify all exon borders. Where possible, EST data has been analysed to help in the annotation process. In addition to the analysis of these large-scale sequencing projects, all WASP family genes in the "nr" database at NCBI have been collected and reanalysed. All sequence related data (names, corresponding species, GenBank ID's, alternative names, corresponding reference publications, domain predictions, and sequences) and references to genome sequencing centers are available through the CyMoBase (http://www.cymobase.org, [66]).

### Generating the multiple sequence alignment

The multiple sequence alignments of the WASP family proteins have been built and extended during the process of annotating and assembling new sequences. The initial alignments have been generated from the first 10 non-validated sequences obtained from NCBI using the ClustalW software with standard settings [67]. During the following correction of the sequences (removing wrongly annotated sequences and filling gaps) the alignment has been adjusted manually. Subsequently, every newly predicted sequence has been preliminary aligned to its supposed closest relative using ClustalW, the aligned sequence added to the multiple sequence alignment of the respective WASP family, and the alignment adjusted manually during the subsequent sequence validation process. Still, many gaps in sequences derived from low-coverage genomes remained. In those cases, the integrity of the exons surrounding the gaps has been maintained (gaps in the genomic sequence are reflected as gaps in the multiple sequence alignment). The unique poly-proline regions are completely divergent in sequence and length and could therefore only be aligned manually. The VCA domains are interrupted by linkers of different lengths, especially in WASH proteins, and were therefore also aligned manually. The sequence alignments of the WASP family proteins can be obtained from CyMoBase, and are also included in Additional file 1.

### Computing and visualizing the phylogenetic trees

The phylogenetic trees were built based on the manually constructed and maintained multiple sequence alignments of the concatenated WASP protein family characteristic N-terminal and VCA (without linker) domains using two different methods: 1. Maximum likelihood (ML) using the PROTMIXBLOSUM62 amino acid model and bootstrapping (1,000 replicates) using RAxML [68]. 2. Posterior probabilities were generated using MrBayes v3.1.2 [69]. Two independent runs with 4,000,000 generations, four chains, and a random starting tree were computed using the mixed amino-acid option. From the 1,000th generation MrBayes exclusively used the JTT model [70] in both runs. Trees were sampled every 1,000th generation and the first 100,000 of the trees were discarded as "burn-in" before generating a consensus tree. Phylogenetic trees were visualized with FigTree (http://tree.bio.ed.ac.uk/software/figtree).

### Availability of supporting data

The data sets supporting the results of this article are included within the article (and its additional files).

## Abbreviations

CRIB: Cdc42/Rac interactive binding; GDB: GTPase binding domain; JMY: Junction-; ediating and regulatory protein; NHS: Nance-Horan syndrome; VASP: Vasodilator-stimulated phosphoprotein; WAHD: WASH homology domain; WAID: WASP autoinhibitory domain; WAML: WASP and MIM like; WASH: Wiskott-Aldrich syndrome protein and Scar homolog; WASP: Wiskott-Aldrich syndrome protein; WAVE: WASP-family verprolin-homologous protein; WAWH: WASP without WH1 domain; WH1: WASP homology domain 1; WH2: WASP homology domain 2; WHAMM: WASP homolog associated with actin, membranes, and microtubules; WHD: WAVE homology domain, WIP: WASP-interacting protein; WMD: WHAMM membrane interaction domain.

## Competing interests

The authors declare that they have no competing interests.

## Authors' contributions

MK assembled and annotated sequences, did all data analysis and wrote the manuscript. DL assembled part of the WASP sequences. SE assembled part of the WAVE sequences. All authors read and approved the final version of the manuscript.

## Supplementary Material

Additional file 1 **Zip archive of the phylogenetic trees and sequence alignments**. The file includes all phylogenetic trees of the WASP family proteins. The sequence alignments of the proteins are included in fasta format. Click here for file
