# Supplementary material for: Evolution of the eukaryotic ARP2/3 activators of the WASP family: WASP, WAVE, WASH, and WHAMM, and the proposed new family members WAWH and WAML
Source: BMC Res Notes. 2012 Feb 8;5:88. doi: 10.1186/1756-0500-5-88 (PMC3298513; doi:10.1186/1756-0500-5-88)

Phylogenetic tree of the WASP proteins

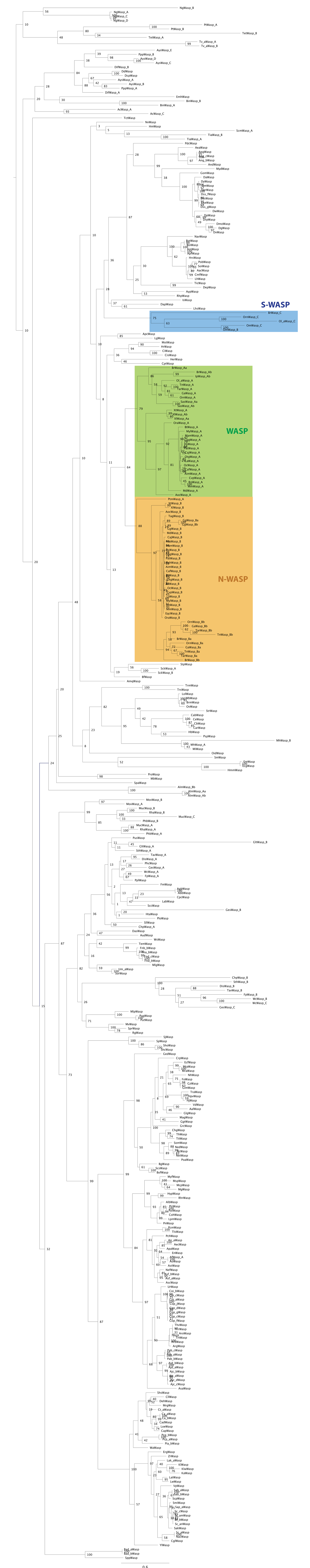

Phylogenetic tree of the WAVE proteins

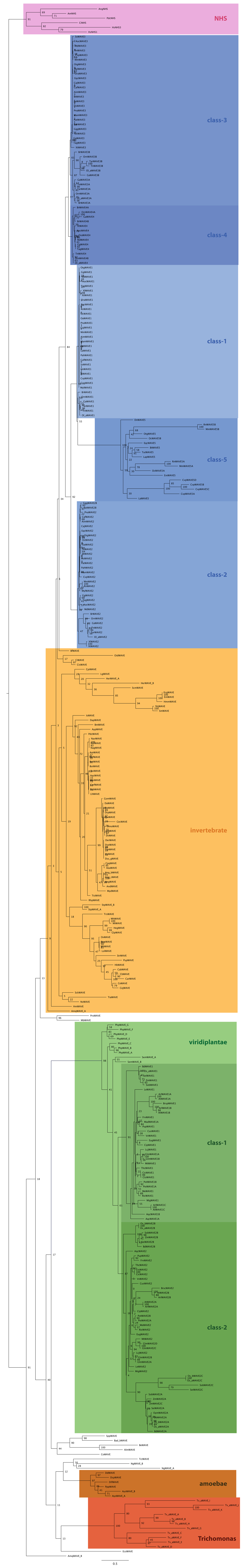

Phylogenetic tree of the WHAMM proteins

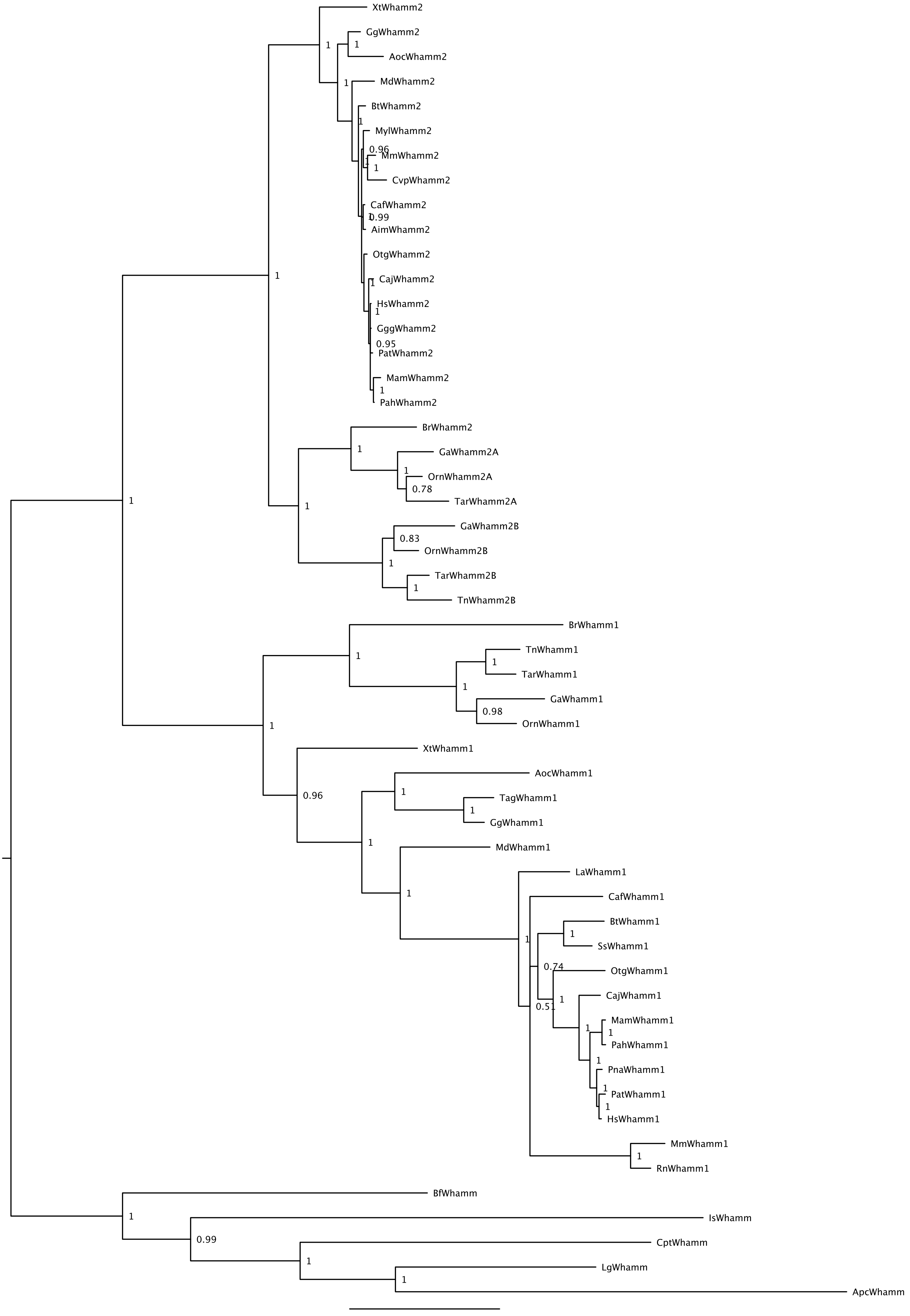

Supplement: Additional file 1 — Zip archive of the phylogenetic trees and sequence alignments. The file includes all phylogenetic trees of the WASP family proteins. The sequence alignments of the proteins are included in fasta format. [file 1756-0500-5-88-S1.ZIP › SupInfo.pdf]
